# Immunomodulation for maxillofacial reconstructive surgery

**DOI:** 10.1186/s40902-020-00249-4

**Published:** 2020-03-05

**Authors:** Seong-Gon Kim

**Affiliations:** Department of Oral and Maxillofacial Surgery, College of Dentistry, Gangneung-Wonju National University, Gangneung, Jukhyun-gil 25457 South Korea

**Keywords:** Immunomodulation, Macrophage, Wound healing, 4-Hexylresorcinol

## Abstract

Immunomodulation is a technique for the modulation of immune responses against graft material to improve surgical success rates. The main target cell for the immunomodulation is a macrophage because it is the reaction site of the graft and controls the healing process. Macrophages can be classified into M1 and M2 types. Most immunomodulation techniques focus on the rapid differentiation of M2-type macrophage. An M2 inducer, 4-hexylresorcinol, has been recently identified and is used for bone grafts and dental implant coatings.

## Background

The maxillofacial area is composed of several different tissues, including the skin, mucosa, bone, skeletal muscle, and salivary glands. Accordingly, different kinds of graft materials are required for maxillofacial reconstructive surgery. Autogenous graft materials have been considered the optimal choice due to the lack of immunological complications [1]; however, not all patients are free from complications. When the tissue is taken from the donor site, blood circulation is blocked, and hypoxic stress is increased. Additionally, some patients with systemic diseases do not have favorable conditions in the recipient site [2]. These situations can lead to graft necrosis and cause immunological complications, such as serious inflammation.

Most types of grafts are successful in otherwise healthy patients. However, the success rate is lower when patients have pre-existing conditions of systemic diseases, such as diabetes mellitus or osteoporosis [3]. Representative examples may be the application of dental implants. Surface modification technology has been developed to improve osseointegration of dental implant in patients with poor bone quality [4]. Dental implants with rough surfaces generally provide greater opportunities for osseointegration as compared to those with the smooth surfaces [5]. Porosity is an important factor in improving the cell survival of bone grafts [6]. Most of the research involving bone tissue engineering have focused on osteoblasts rather than immune cells such as macrophages [7]. This review may provide a foundation for immunomodulation research.

A recent review summarized the current understanding of immunomodulation in bone grafts or osteoimmunomodulation [8]. Most strategies implementing osteoimmunomodulation have been confined to the modulation of pore- or particle-sized grafts and the change of surface properties. Though these simple modifications have shown success in dental implants [9, 10], the precise molecular mechanism has not been clarified. The modification of composition may demonstrate a more advanced level of immunomodulation. However, the addition of nutrient elements, such as magnesium or silicate, does not seem to show significant improvement as compared to the modification of physical properties. Some cytokines or bacterial toxins, such as lipopolysaccharide, are possible ingredients for immunomodulation of bone grafts [11, 12]. However, most protein-based ingredients exacerbate inflammatory responses and have side effects that are difficult to control. To improve the controlled release of ingredients, smart drug carriers have been developed that are designed to release active ingredients as intended [13].

These efforts have dramatically improved the effectiveness of immunomodulation and the design of the drug carriers. However, the fundamental mechanism between biomaterial and host response is not well understood. Particularly, the wound healing mechanism is largely unknown. The response to stress-mediated mitochondria may be a key element for understanding these mechanisms. The purpose of this review was to summarize the current level of understanding of macrophages in immunomodulation. Therefore, the application of 4-hexylresorcinol (4HR) in immunomodulation was reviewed in detail.

## Main text

### Classical approach for immunomodulation

#### Immunomodulation by changing physical property

##### Surface roughness

**Fig. 1:**
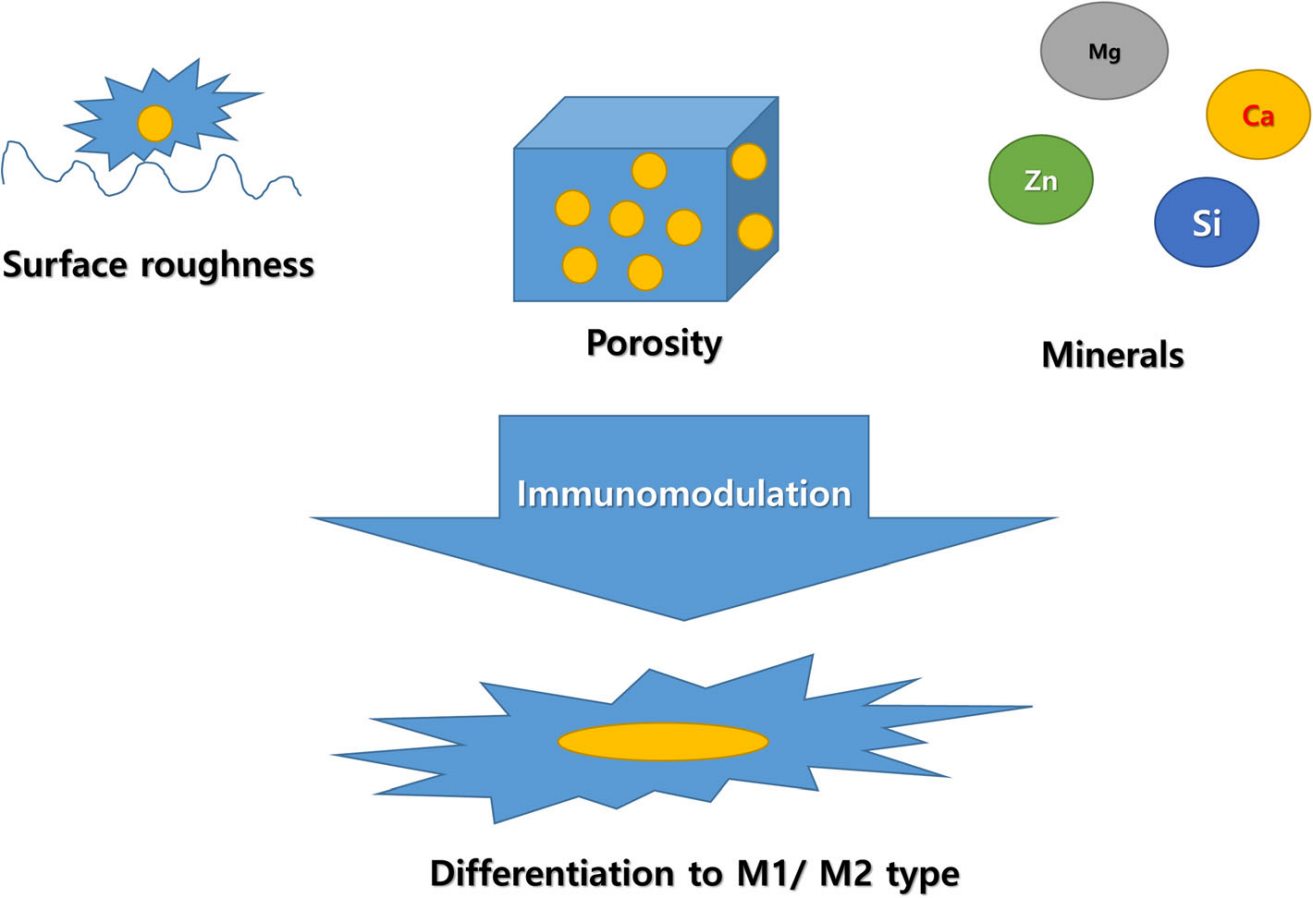
Classical way of immunomodulation. The immunomodulation technique can be applied to achieve a desirable host response to grafts. Modification of surface roughness and porosity is a simple method for immunomodulation. Some trace elements have also been considered for helping wound healing

When a graft is implanted into the body, cells attach to the graft surface. Cell response to surface roughness (Fig. 1) has been widely studied in the field of dental implants. Dental implants are installed into the alveolar bone, and the success of osseointegration is dependent on the surface texture of the dental implant. Generally, rough-surfaced implants show better osseointegration as compared to smooth-surfaced implants [5]. As a modification of surface roughness, secretion cytokines favorable to healing are increased by the macrophages [14].

Bone grafts are designed to imitate the surface roughness of the natural bone with 32 nm grain sizes [15]. Many techniques have been developed to increase the surface roughness of dental implants, which includes sandblasting and acid etching techniques [14]. Dental implant surfaces with M1 polarization impair osseointegration [16]. However, the modification of surface roughness can activate both M1 and M2 types of macrophages and cannot activate any specific type [17].

It is unclear whether hydrophobic or hydrophilic surfaces are better for surface wettability. Hydrophobic surfaces show more intense monocyte-derived immune reactions than hydrophilic surfaces [18]. Also, hydrophilic surfaces are not favorable to macrophage attachment [19]. However, the production of pro-inflammatory cytokines is greatly enhanced on hydrophilic surfaces [20].

##### Porosity

The porosity of the graft is closely associated with oxygen concentration and nutrient supply [9]. Also, highly porous grafts may provide a better opportunity for cellular migration [6]. One study demonstrated that pore size also affects angiogenesis, which is delayed when the pores are smaller (90–120 μm as compared to 300 μm) [21]. Bone graft material with 80–88% porosity is better for osteogenesis [6].

#### Immunomodulation by adding minerals

The addition of minerals has been used for the development of bone grafts. The bone is mainly composed of hydroxyapatite; therefore, hydroxyapatite-based grafts or coatings have been used for orthopedics and dental implants [22, 23]. Calcium is a major component of hydroxyapatite, and the effect of calcium is concentration-dependent. Calmodulin-dependent kinase responds to elevated concentrations of calcium ions and activates the nuclear factor-kappa B (NF-κB) pathway and increases inflammation [24]. However, high concentrations of extracellular calcium ions inhibit the NF-κB pathway via Wingless-int (Wnt) 5A [25].

Many types of trace elements have been used as ingredients for bone grafts. Silicon-based grafts show osteoblast activation [26], and higher concentrations of silicon also suppress osteoclastic activity [27]. Zinc can also increase osteogenesis [28]. Cobalt can be added to promote angiogenesis [29]. Magnesium is added to improve the biodegradability of the graft [30]. Magnesium ions can suppress host immune responses via the inhibition of the toll-like receptor (TLR) signaling pathway [31].

### Macrophage and angiogenesis

**Fig. 2:**
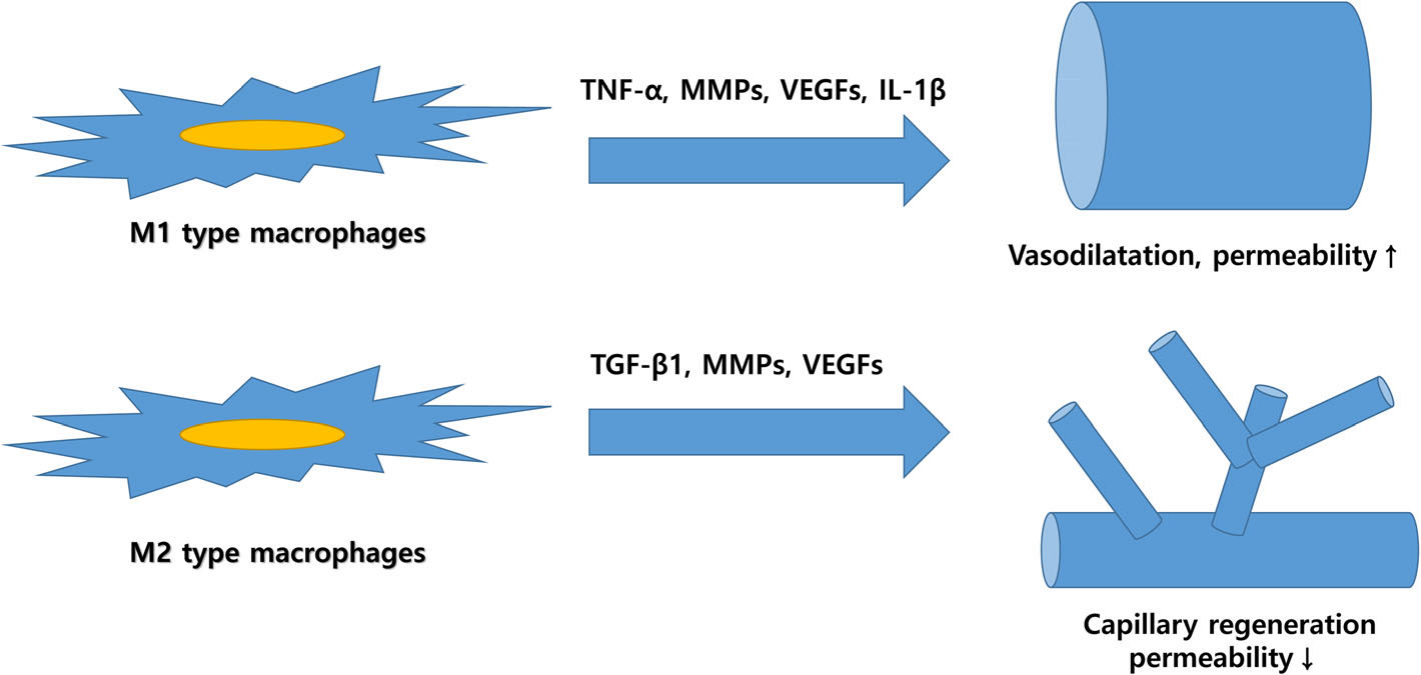
Role of M1/M2-type macrophages in angiogenesis. M1-type macrophages are responsible for host defenses again invasion. Thus, cytokines secreted from M1-type macrophages recruit leukocytes through vasodilatation and increasing vascular permeability. However, M2-type macrophages are responsible for regeneration. Accordingly, they secrete cytokines for capillary regeneration. Some cytokines are overlapped between M1 and M2 macrophages, and their final role is determined by the interaction with other cytokines

Appropriately, timed angiogenesis is essential for normal wound healing, (Fig. 2). Some complications, such as diabetic retinopathy, are caused by impaired angiogenesis [32]. One possible reason of medication-induced jawbone necrosis is impaired angiogenesis after oral surgery [33]. According to a recent publication, the macrophage is a key cell for the healing process and enhances neovascularization via the M1-to-M2 transition [34].

M1-type macrophages are predominant in the inflammatory phase and secrete pro-inflammatory cytokines [35]. The roles of pro-inflammatory cytokines include vasodilation and chemotaxis for recruiting more leukocytes to the wound region [36]. However, M2-type macrophages are predominant in the healing phase. M1-type and M2-type macrophages are generally predominant 1–5 and 4–10 days after grafting, respectively [37, 38]. The precise mechanism for the M1-to-M2 transition is largely unknown. According to our recent research, antioxidants such as 4HR can induce M2-type macrophages and accelerate angiogenesis [39]. Sequential administration of interferon-gamma (pro-inflammatory cytokine) and interleukin-4 (M2 inducer) increases vascularization of the bone graft [34]. These administrations are redundant and can be simplified, as some inflammation is unavoidable when any graft material is implanted [40]. Thus, presence of an M1 inducer in the graft may not be necessary.

### Many types of cytokines and toxins can induce M1/M2 phenotypes

**Fig. 3:**
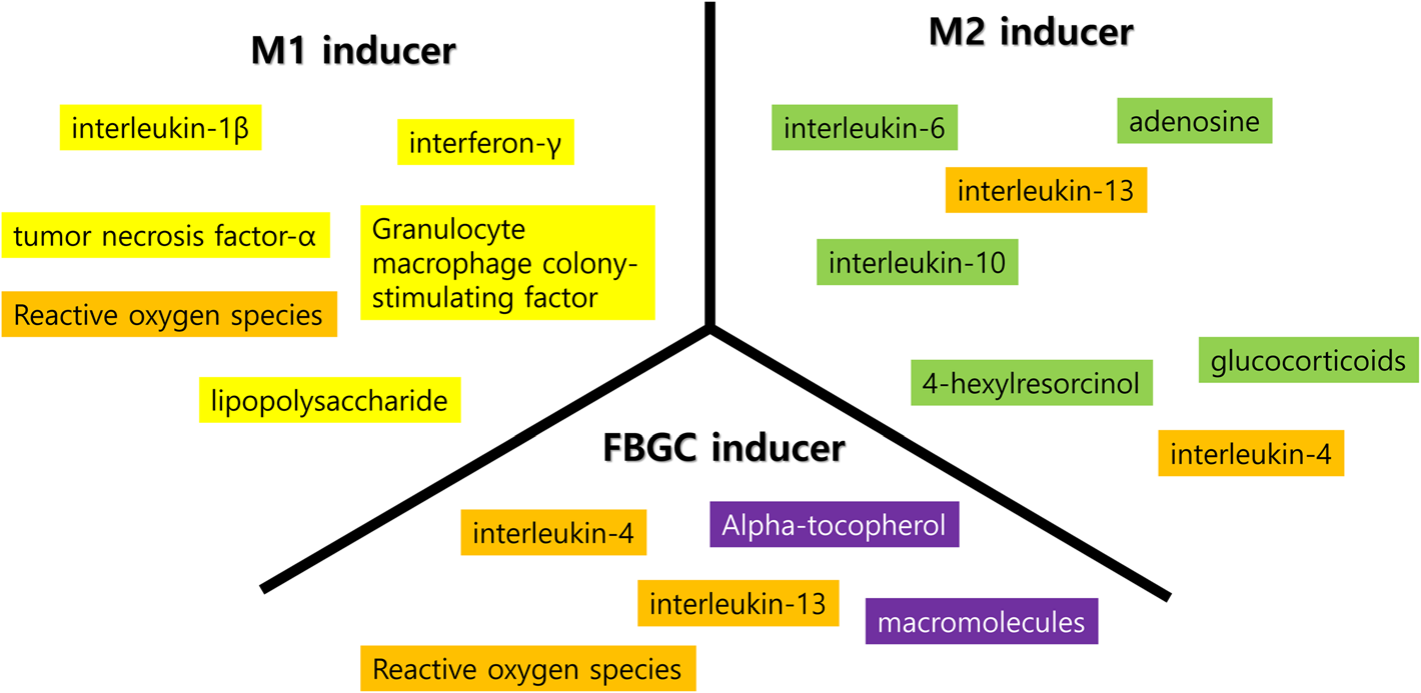
M1/M2/foreign body giant cell (FBGC) inducers. There are many kinds of M1/M2/FBGC inducers. Some of them are not determinant, and the outcome is influenced by the environment

Macrophage phenotypes, such as M1, M2, or multinucleated giant cells, may be the consequence of survival strategies in response to environmental changes (Fig. 3). Foreign body giant cells (FBGCs) are considered a sign of graft rejection [19]. FBGCs exhibit inflammation associated with cytokines, such as interleukin-1β (IL-1β) and tumor necrosis factor-α (TNF-α) [19]. However, some studies show that FBGCs express M2 markers [41]. Accordingly, FBGCs may have M1- and M2-type macrophages, including single nuclear macrophages. The body considers grafts to be foreign materials, and an immune reaction is unavoidable. During cellular digestion, some resistant polymers may not be degraded quickly because of size [42], and these materials cannot be digested by a single macrophage. Slower degradation increases reactive oxygen species (ROS), which can induce apoptotic stress in mitochondria. For the survival of macrophages in this environment, increasing the volume of cytoplasm may be an effective strategy for reducing ROS concentrations. Fused macrophages still attempt to degrade grafts [43]; however, fused macrophages show lower enzyme activity as compared to the sum of the unfused group [44]. Lower enzyme activity and diluted ROS are beneficial for macrophage survival from apoptotic pressure. When macrophage apoptosis occurs, the FBGC formation is decreased [45]. Some M2 markers respond to cytoplasmic ROS levels, and the expression of these markers in FBGCs seem to be the consequence of cellular fusion. Therefore, M2 marker expression in FBGCs may not be an indicator of normal healing. The artificial fusion of macrophages can be induced by the application of interleukin-4 and interleukin-13 [46].

M1 inducers, such as interferon-γ (IFNγ), lipopolysaccharide (LPS), and TNF-α [47], may be required for macrophages to differentiate into the M1 type. Macrophages have many receptors for the detection of foreign materials, such as toll-like receptors [48]. The activation of these receptors induces M1-type macrophages, and M1 macrophages secrete anti-microbial or degrading enzymes [49]. Known M2 inducers are several interleukins including IL-4, IL-10, IL-13, steroid hormones, and 4HR. M2 macrophages are classified as M2a, M2b, and M2c; however, the specificity for these M2 inducers has not been studied extensively. IL-4 and IL-13 are known to induce M2a [50]; however, IL-4 can induce multinucleated giant cells as well [51]. Macrophages are highly responsive to environmental demand, and therefore, their phenotype is highly flexible and reversible [52, 53].

### Immunomodulation in wound healing

Following a tissue injury, the epidermis can regenerate, but scar-free healing is still unattainable for skin wounds. Systemic diseases associated with the host immune system and aging may result in impaired wound healing [54, 55], which are related to impaired transitions from M1- to M2-type macrophages [55]. Few organs show true tissue regeneration, and most are regenerated with dense fibrous tissue [56]. Macrophages secrete many kinds of cytokines such as transforming growth factor-β1 (TGF-β1), vascular endothelial growth factor (VEGF), and matrix metalloproteinases (MMPs), and they can activate fibroblasts [57].

**Fig. 4:**
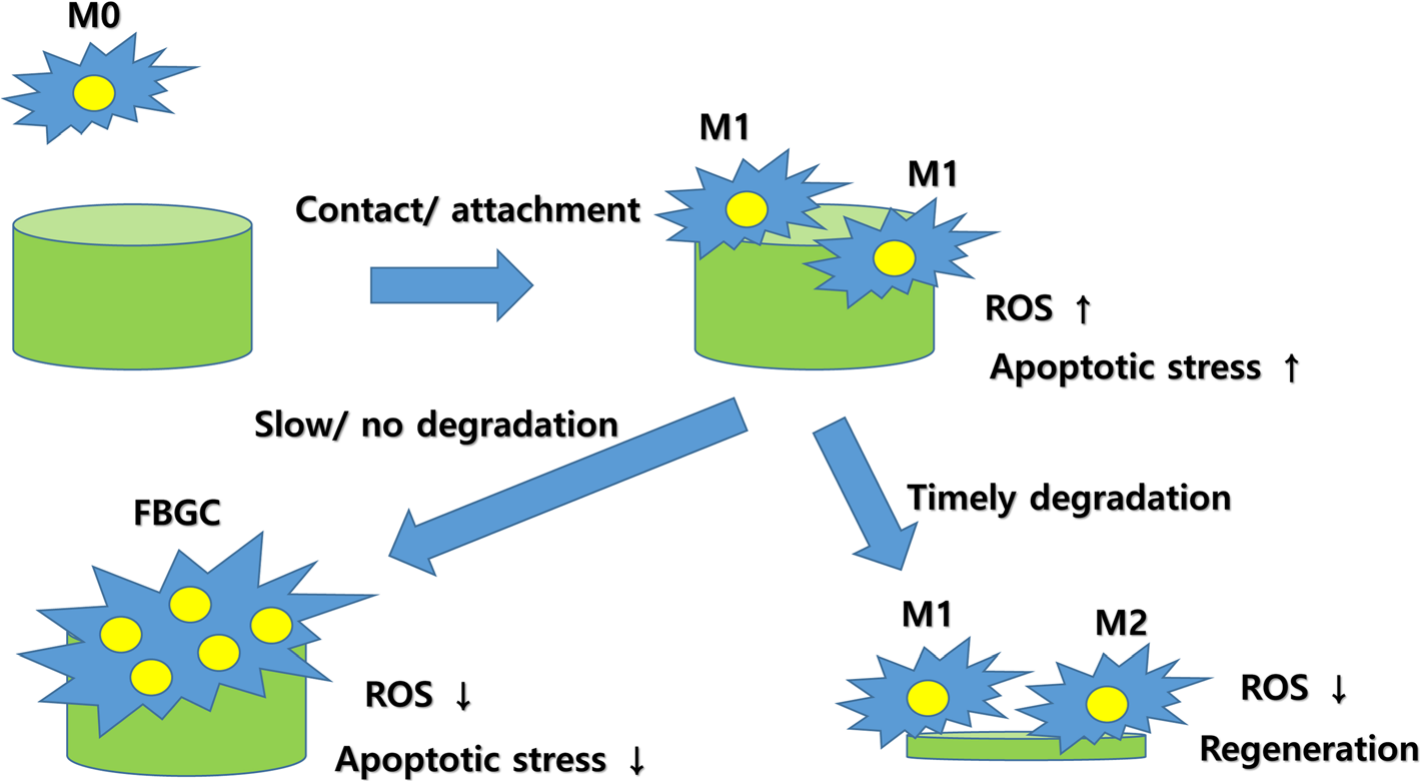
Hypothesis for M1/M2/foreign body giant cell (FBGC) formation. Cellular stress induced by a foreign body may be the driving force for macrophage differentiation. Reactive oxygen species (ROS) may also be a key factor for macrophage differentiation. Slow or nondegradable grafts accumulate stress in the macrophages. To reduce intracellular stress, increasing the volume of cytoplasm via cellular fusion can be a useful strategy for survival. If the graft is degraded appropriately, intracellular stress is reduced thereafter. M2 transition will occur after reduced stress

There have been several hypotheses for macrophage differentiation. Resident macrophages in most organs are the first line of defense against foreign bodies [58]. Some tissue-resident macrophages may have stem cell-like abilities [59]. When an injury occurs, these macrophages, which are generally M1 type, increase their population and recruit immune cells from the blood [58]. The cytokines expressed by M1-type macrophages are cytotoxic [60]. M1-type macrophages produce ROS and are associated with the immune response against microorganisms [40]. Increased levels of ROS are closely related to apoptosis and phase transformation to either M2-type macrophages or FBGCs. The formation of giant cells is an assumed survival strategy of M1-type macrophages; therefore, FBGCs should produce low levels of ROS [44]. Proliferation and differentiation are mutually exclusive cell developmental stages. The proliferation dominant stage is the inflammatory phase, and its duration is limited by many factors, including the virulence of invading microorganisms, the number of functional immune cells, and the supply of oxygen and nutrients. The interaction among these factors determines the duration of the inflammation [40]. When the cell defense is successful, a disproportionate number of macrophages remain, which leads to apoptotic stress in the macrophages. ROS is mainly produced by M1-type macrophages and induces cell death [61]. Some macrophages undergo apoptosis, while others survive and differentiate into M2-type macrophages. In this stage, most newly visiting macrophages also differentiate into M2-type macrophages. Apoptotic stress can be induced when grafts are too large for digestion. As a survival strategy, macrophages can be fused (Fig. 4). Wound is isolated by macrophages and fibroblasts with dense fibrotic tissue as defense mechanisms [62]. If isolation occurs, the inside of an infection is transformed from acute to chronic, which is unstable. Accordingly, both M1- and M2-type macrophages may be present in the chronic inflammation. As a consequence, both pro- and anti-inflammatory cytokines are found together [40].

### 4-Hexylresorcinol for the immunomodulation

**Fig. 5:**
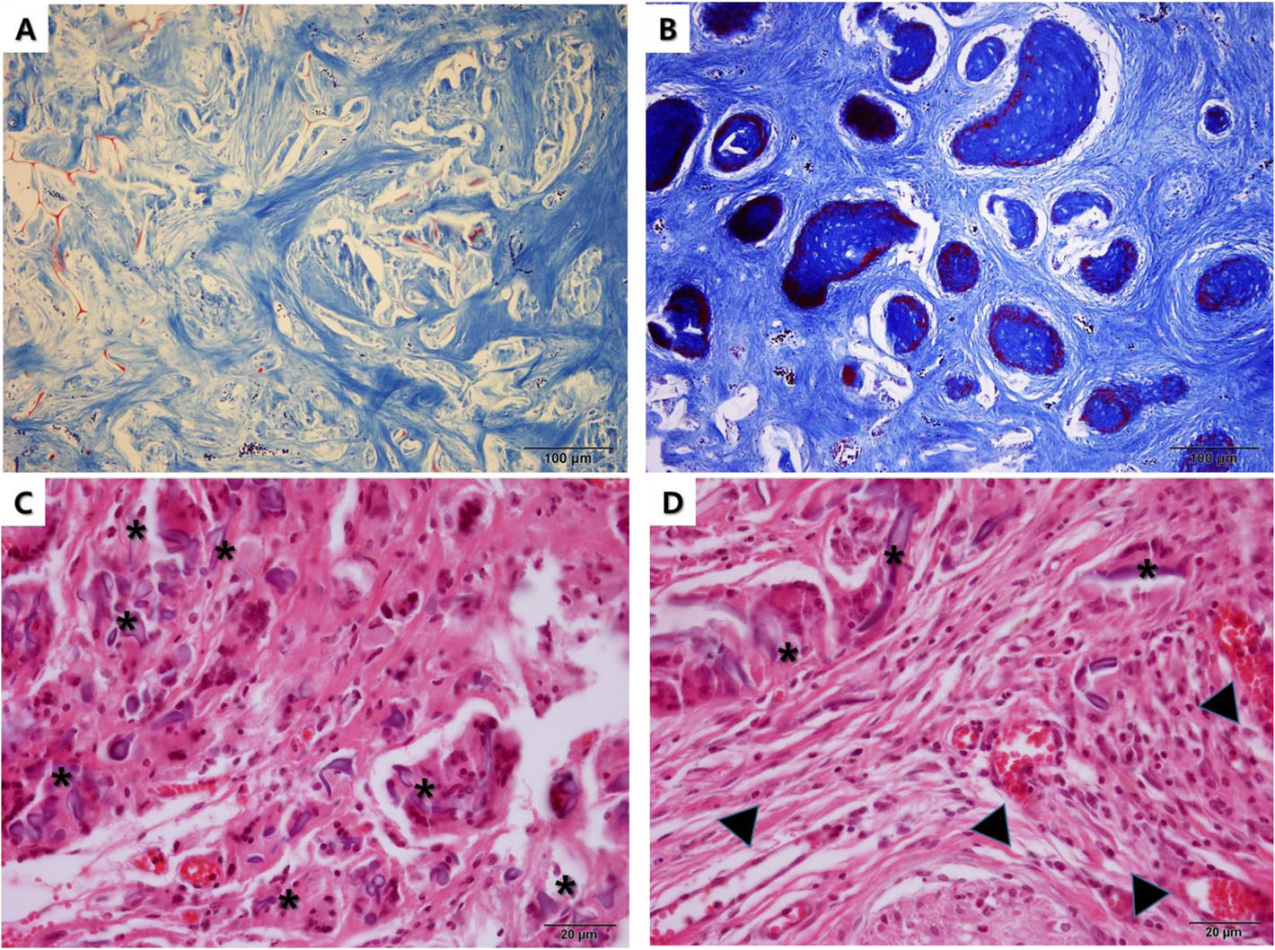
Accelerated tissue regeneration by incorporating 4-hexylresorcinol (4HR). **a** Silk and hydroxyapatite grafts were implanted into calvarial defects of rabbits. There was no bone regeneration at 8 weeks postoperation (Masson trichrome stain, original magnification ×100). **b** A silk fibroin, hydroxyapatite, and 4HR graft were implanted into the calvarial defect of a rabbit. There were numerous osteoid islands at 8 weeks postoperation (Masson trichrome stain, original magnification ×100). **c** A silk fibroin graft was implanted into the dermal pocket of a rat. There were many foreign body giant cells (asterisks) with silk fiber remnants at 12 weeks postoperation (HE stain, original magnification ×400). **d** A silk fibroin and 4HR graft were implanted into the dermal pocket of a rat. There were few foreign body giant cells (asterisks) at 12 weeks postoperation. Interestingly, capillary regenerations were prominent (arrow heads) (HE stain, original magnification ×400)

4HR is an emerging material for use in immunomodulation (Fig. 5). 4HR administered to M2-type macrophages expresses a high level of TGF-β1 [63, 64]. Osteal macrophages (OsteoMacs) produce osteoblast activators, such as TGF-β [65], osteopontin [66], and bone morphogenetic protein 2 (BMP-2) [67]. Bone regeneration is significantly impaired without OsteoMacs [68]. The coupled reaction between osteoblasts and osteoclasts and TGF-β produced by macrophages are essential for bone remodeling [69, 70]. Generally, M2-type macrophages, including M2a, express TGF-β [35, 71]. Anti-inflammatory M2b- and M2c-type macrophages usually express IL-10 [72]. Recently, M2d-type macrophages were introduced and found to secrete TGF-β, VEGF-A, and TNF-α [73]. M2d-type macrophages are associated with wound healing [73]. When a dental implant is coated with hydroxyapatite with 4HR, bone formation around the implant surface is enhanced [74].

However, function-based discrimination among M2 subtypes is difficult. The spectrum of expressed markers overlaps. Any inducer of M2-type macrophages is considered to be beneficial for graft success [75]. 4HR inhibits the NF-κB pathway [76] and the expression of TNF-α [77]. Accordingly, 4HR-induced M2-type macrophages have M2d characteristics except for TNF-α expression. The limitation of current research regarding 4HR is that most cellular experiments have been conducted using murine macrophages. Since there is a considerable difference between human macrophages and murine macrophages [78], more specific studies on human macrophages should occur.

4HR can increase TGF-β1 expression in human umbilical vein endothelial cells (HUVEC) (data submitted for publication) and can also increase VEGFs mediated by TGF-β1 (data submitted for publication). TGF-β1 is an important cytokine in wound healing [79]. Though the downstream signals from TGF-β1 are divergent, 4HR decreases TGF-β1 associated inflammatory reactions by inhibiting the NF-κB pathway [63]**.** In the diabetic animal model, 4HR ointment showed a substantial increase in capillary regeneration as compared to the untreated control (data submitted for publication).

### Role of smart material in the immunomodulation

Controlled release is ideal for optimizing drug effects and minimizing potential side effects. Smart material is designed for controlled release [80]. One example of smart material is pH-responsive polymers. Inflammatory tissue has a lower pH as compared to the surrounding normal tissue; therefore, low pH-responsive drug carriers could be used as an optimal anti-inflammatory response. Conversely, any per os medication may be protected from degradation caused by gastric acid.

**Fig. 6:**
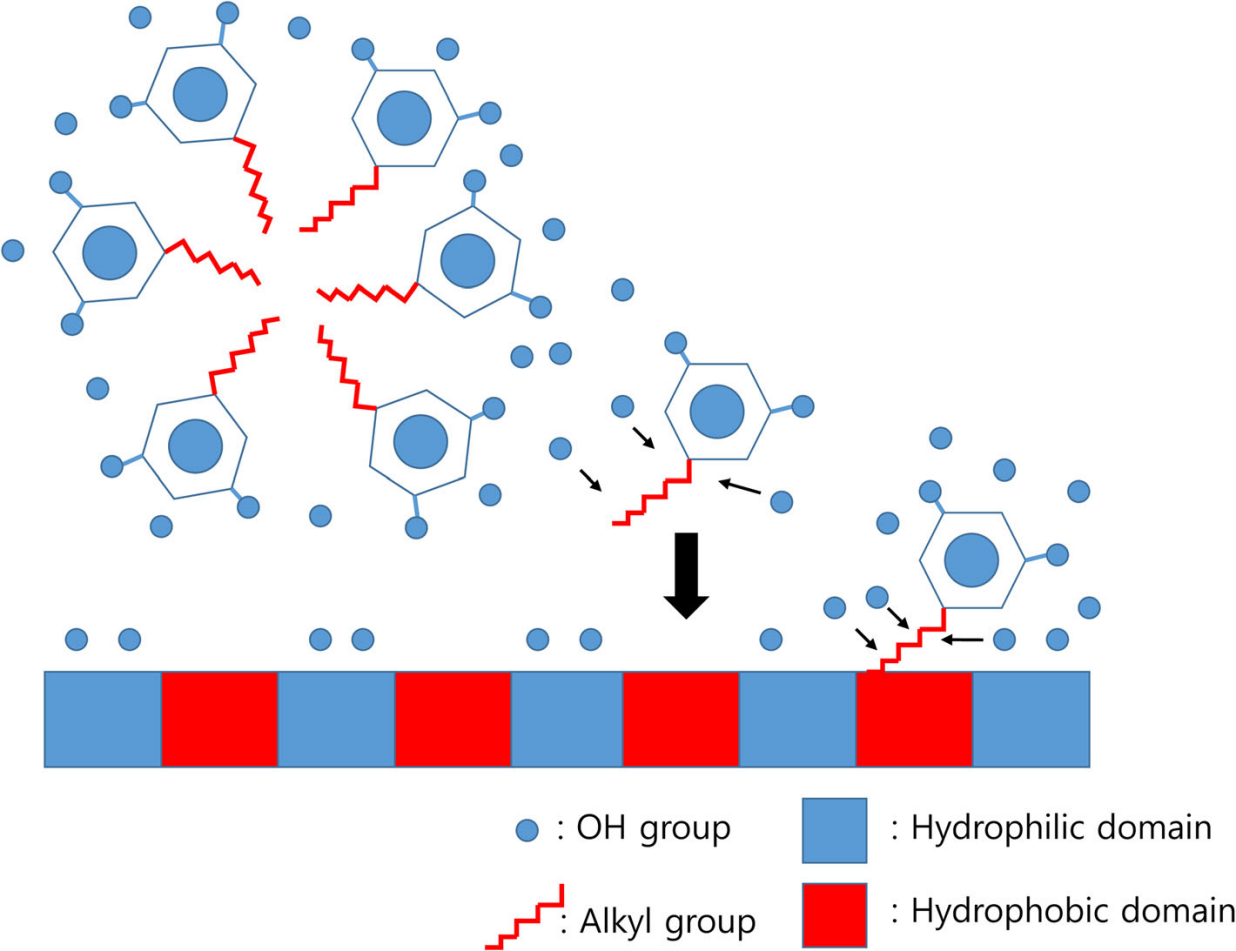
Smart delivery of 4-hexylresorcinol (4HR) using hydrophobic and hydrophilic domains of silk fibroin. Silk fibroin is a macromolecule composed of hydrophilic and hydrophobic domains. As 4HR is composed of a long alkyl group (hydrophobic) and 2 hydroxyl groups (hydrophilic), 4HR can be bound to each domain according to its molecular interaction. As hydrophilic interactions are much weaker than hydrophobic interactions, 4HR in the hydrophilic domain of silk fibroin is released first. 4HR in the hydrophobic domain is released during proteolysis

The modification of physical properties is valid only in the early stage of grafting. After initial contact with host cells, the effects on wound healing are limited. However, when active components are released early enough, their effect is observed throughout all healing stages (Fig. 6). Many types of synthetic materials demonstrate slow biodegradation. When these materials are grafted, dense fibrotic tissue is formed within 2–4 weeks [81]. Dense fibrotic tissue has low vascularity, which hampers further degradation. Accordingly, the modification of pore size and shape has been utilized to induce angiogenesis and suppress fibrosis [82]. The most biodegradable polymer has undergone hydrolysis [80, 83], which accounts for the discrepancy between in vitro and in vivo degradation test results. Though the human body is mainly composed of water, slowly degraded polymer is surrounded by dense fibrotic tissue. Dense fibrotic tissue prevents water exchange. Therefore, it is different to in vitro condition such as dipping into water. In our experience, many kinds of biodegradable plates were still found years after installation [84].

Natural macromolecules can be substitutes for synthetic polymers. The collagen-based matrix has been widely tested and used. Collagen is the main component of the extracellular matrix. Scaffold mimics may have an advantage in the transition of macrophage type [85]. Silk fibroin is also used as a drug carrier for controlled release [86]. Compared to the collagen matrix, silk fibroin is a slowly degraded material. Accordingly, the drug release speed of silk fibroin carriers is slower than that of collagen carriers. Based on the target organ and speed of healing, optimal drug carriers can be selected. As 4HR can accelerate the proteolysis of silk fibroin in a concentration-dependent manner, the drug release speed of the silk fibroin carrier can be modified by adding 4HR.

## Conclusion

Wound healing is influenced by the general health of the patient. To maximize graft success rates, immunomodulation is essential. Many kinds of immunomodulation have been used and studied in maxillofacial reconstructive surgery. 4HR is a new M2 inducing material that can be applied in various fields of tissue engineering.

## Data Availability

Not applicable.

## Abbreviations

4HR: 4-Hexylresorcinol
FBGC: Foreign body giant cell
HUVEC: Human umbilical vein endothelial cell
IFNγ: Interferon-γ
IL-1β: Interleukin-1β
LPS: Lipopolysaccharide
MMP: Matrix metalloproteinase
NF-κB: Nuclear factor-kappa B
OsteoMacs: Osteal macrophages
ROS: Reactive oxygen species
TGF-β1: Transforming growth factor-β1
TLR: Toll-like receptor
TNF-α: Tumor necrosis factor-α
VEGF: Vascular endothelial growth factor
Wnt: Wingless-int
